# Assessing the impact of digital education and the role of the big data analytics course to enhance the skills and employability of engineering students

**DOI:** 10.3389/fpsyg.2022.974574

**Published:** 2022-10-19

**Authors:** Lin Xu, Jingxiao Zhang, Yiying Ding, Gangzhu Sun, Wei Zhang, Simon P. Philbin, Brian H. W. Guo

**Affiliations:** ^1^School of Foreign Languages, Northwest University, Xi’an, China; ^2^School of Economics and Management, Chang’an University, Xi’an, China; ^3^School of Civil Engineering, Zhengzhou University, Zhengzhou, China; ^4^Institute of China’s Science Technology and Education Policy, Zhejiang University, Hangzhou, China; ^5^School of Engineering, London South Bank University, London, United Kingdom; ^6^Department of Civil and Natural Resources Engineering, University of Canterbury, Christchurch, New Zealand

**Keywords:** digital education and training, big data analytics courses, engineering student employability, hard skills, soft skills

## Abstract

This study aims to explore the role of digital education in the development of skills and employability for engineering students through researching the role of big data analytics courses. The empirical study proposes the hypothesis that both soft and hard skills have positive effects on human capital, individual attributes, and the career development dimensions of engineering students. This is achieved through constructing a framework of three dimensions of engineering students’ employability and two competency development dimensions of big data analytics courses. A questionnaire survey was conducted with 155 college engineering students and a structural equation model (SEM) was used to test the hypotheses. The results found that courses on big data analytics have a positive impact on engineering students’ abilities in both hard skills (*p* < 0.01) and soft skills (*p* < 0.001) dimensions, while soft skills have a more significant impact on engineering students’ employability. The study has practical and theoretical implications that further enriches the knowledge base on engineering education and broadens our understanding of the role of digitalization in enhancing the skills and employability of engineering students.

## Introduction

The employability of graduates plays a crucial role in the development of individuals, including their careers, as well as having a wider impact on economic growth. Indeed, employability is a multidimensional concept related to the ability of individuals to enter or remain in employment (Mackieh and Dlhin, 2019). This has been an important outcome for higher education institutions since employers are key stakeholders for the education process. With the rapid development of the information society, industry has placed higher demands on the employability of engineering graduates (Winberg et al., 2020). In our complex society, having a well-trained engineering workforce is an essential condition for technological progress and economic growth. However, survey data from different countries identifies that there is a recognized shortage of engineers worldwide (Saidani et al., 2022). Moreover, the results of studies on the attributes of engineering students show that engineering graduates generally complete their education with a lack of knowledge related to employability and wider professional skills (Koka and Raman, 2015).

It can be observed that many employers perceive graduates as not being prepared for the workplace and graduating students lack many of the essential skills needed for successful employment, such as communication, teamwork, resilience and critical thinking skills (Rizwan et al., 2018). The main reasons for the lack of these employability skills are as follows:

1. There is a huge discrepancy between engineering graduates’ perceptions of employability and employers’ expectations, with employers tending to consider communication skills, interpersonal skills, creativity and critical thinking to be more important. While graduates often believe that their own vocational and technical skills are more likely to help them find a job (Rizwan et al., 2018);
2. The current educational system still follows a structured and memory-based curriculum, resulting in a lack of creative thinking and problem-solving opportunities, which can reduce the impact of students in the current job market (Rizwan et al., 2021);
3. In terms of bridging the gap between higher education and industry, developed countries are able to provide engineering students with opportunities to acquire first-hand insights, materials and competencies from the workplace. This is achieved through industry placements, research projects and other incentives to expose students to the workplace, while developing countries are still lagging behind in the implementation of such initiatives (Puni et al., 2018).

Higher education is considered to be one of the most important factors affecting the quality of employment (Lee and Chung, 2015). Indeed, engineering students at higher education institutions are able to acquire the underpinning competencies needed for employment, secure in-depth expertise, and learn the ability to transfer knowledge from the scientific and academic fields to the professional workplace (Brennan and Teichler, 2008). Therefore and in recent years, higher education institutions have been increasingly called upon to develop the employability of engineering students for improved access to the graduate job market. However, traditional engineering curricula do not always promote meaningful learning and often do not motivate students to take responsibility for their own learning outcomes (Chand et al., 2019). In this context, the employability of engineering students can be severely compromised. Therefore, in order to integrate students into the job market faster and better, higher education institutions have the responsibility to optimize educational mechanisms and change the traditional pedagogical model to improve the employability of engineering students and help prepare them for employment (Pauceanu et al., 2020).

In recent years, rapid developments in the field of science and technology have led to significant changes in the process of information production, sharing and communication. Such changes have increased the use of information and communication technologies (ICT) in the field of education, thereby contributing to the spread of digital educational environments (Mishra et al., 2020). Digital training brings the following impacts to modern education:

1. Educators can integrate traditional teaching methods when conducting digital training, which positively impacts the effectiveness of teaching and learning (Joshi et al., 2022).
2. Educators are able to observe and assess each student’s progress at different stages of the educational cycle through digital networks and software. More immediate and authentic assessment of learning outcomes creates a unique learning experience for students, thereby allowing them to reflect on their personal knowledge and practices, and improve learning effectiveness (Woods, 2020).
3. Online communication is more focused on the purpose of the task and the immediacy of the communication, and it can in some cases quickly build trust between educators and students, making it an effective educational mechanism to promote collaborative learning (Ugur and Turan, 2018).
4. Digital networks provide learners with powerful tools to retrieve information more efficiently and conveniently, thereby facilitating the evolution of students’ learning styles. In addition, digital systems develop students’ ability to combine traditional subject core competencies with information technology competencies, as well as to improve their ability to capture and critically evaluate information in the rich online knowledge base of the present time (Marcial and Arcelo, 2016).

In conclusion, digital enabled education can be viewed as an emerging strategy to promote reform of the educational system, so as to enhance the education of engineering students and lead to improve employability. Consequently, it is necessary to strengthen the development and popularization of digital training in engineering education and promote the corresponding training mechanism in regard to modernization and digitalization (Garzon Artacho et al., 2020). Therefore, the research question of this study is: What role does digital education play in the employability development of engineering students? Also, how does it work? The study takes the big data analytics course as the object, which is an important part of digital training, as an entry point to study its impact on the employability of engineering graduates.

This study proposes the hypothesis that both soft and hard skills developed in big data analytics courses have a positive impact on the employability of engineering students, and aims to explore the role of digital education on the employability of engineering students. From a theoretical perspective the study broadens our understanding of the employment development pathway for engineering graduates and enriches the theoretical research on engineering education. From the practical perspective, the study strengthens the relationship between engineering education and digitalization; promotes engineering education in the digital era; improves the employability of engineering students and career development through hard skills (such as analytical skills and technical skills), and soft skills (such as critical thinking and innovation) developed in the big data analytics course. Moreover, engineering students are able to have a clearer understanding of the competencies required for engineering employment with the help of digital training.

## Literature review

### Employability dimension

Employability is defined as a set of achievements that make graduates more likely to secure employment and succeed in their chosen career (Yorke, 2004). In this study, we searched the mainstream databases with “employability” as the keyword, and further sifted through the relevant literature focusing on the specific connotation of employability, and finally selected nine representative sources of literature to analyze and summarize the dimensions and classifications of employability. Table 1 shows that employability can be divided into four dimensions, namely human capital, social capital, individual attributes, and career development.

**Table 1:** Employability dimensions.

Human capital	Social capital	Individual attributes	Career development	Reference
Human capital	Social capital	Personal adaptability	Career identity	Fugate et al. (2004), Gedye and Beaumont (2018)
Possession	Position		Process	Holmes (2013)
Human capital	Social capital	Individual attributes	Individual behaviors	Clarke, (2018), Greer and Waight (2017)
Job-related expertise and attitudes	Career-related employability capital (including social capital)		Development-related employability capital	Peeters et al. (2019), Singh et al. (2017)
Human capital	Social capital	Lifelong learning and flexibility	Reflection on self and organization	Römgens et al. (2020), McQuaid and Lindsay (2005)

Table 1 shows the connotations of the four dimensions of employability, which were identified through reviewing the employability-related literature.

The term “human capital” is explicitly used by several authors (Fugate et al., 2004; Clarke, 2018; Römgens et al., 2020). According to Römgens et al. (2020), the human capital dimension refers to the knowledge, skills and attitudes required to meet performance expectations in a given occupation or professional field. Whereas Fugate et al. (2004) define human capital as a set of factors that influence the likelihood of one’s career development, such as age, education, cognitive ability, emotional intelligence and work experience. In addition, this dimension includes Holmes’ (2013) use of the concept of possession (i.e., the skills and attributes of graduates that are seen as being capable of being possessed and used) as well as work-related expertise and attitudes according to the study by Peeters et al. (2019). Combining the views of different researchers, it is evident that human capital refers to the abilities, occupational expertise, work experience, knowledge, skills and attitudes that a person needs to possess in order to perform effectively in his or her current job.

According to Römgens et al. (2020), social capital refers to the knowledge, skills and attitudes embedded in relevant social networks and the development of relevant work relationships. Therefore, we can view this in the context of what Peeters et al. (2019) refer to as “career-related employability capital” (i.e., personal resources that enable individuals to transition between jobs and organizations and gain new labor market positions). These competencies are also included in the definition of the social capital dimensions of employability by Fugate et al. (2004), Clarke (2018) and Römgens et al. (2020) with respect to networks, social class, university ranking, and social competence.

According to Clarke (2018), personal attributes include personality variables, adaptability and flexibility. All of these are considered to be the basis for professional success (Fugate et al., 2004). Among them, adaptability and flexibility help individuals to cope with constant change, while adapting and optimizing helps individuals to prepare for future work challenges, which can lead to successful career outcomes (Heijde and Van Der Heijden, 2006). Personality variables remain relatively stable throughout life, but they can encourage individuals to have an adaptive and flexible attitude toward job searching as well as the acquisition of key career-related skills (Clarke, 2018).

**Figure 1:**
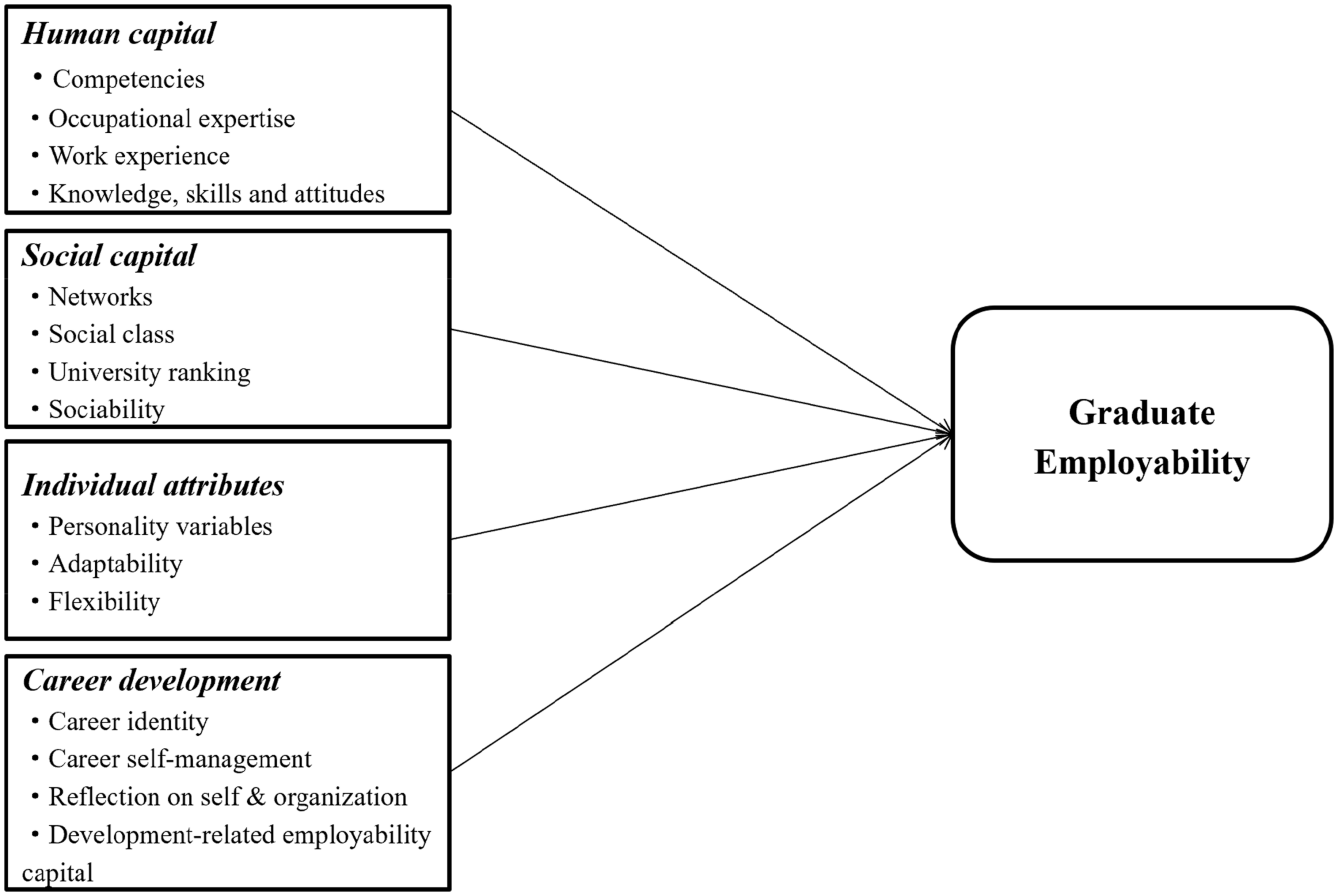
Connotation of employability dimension.

A further dimension of personal attributes can be viewed as career development. This is about the personal qualities that support the sustainability of an individual’s career. Thus, it encompasses career identity (Fugate et al., 2004), personal behavior (i.e., career self-management and career-building skills; Clarke, 2018), developmentally relevant employability capital (i.e., personal resources that can grow over time; Peeters et al., 2019), and reflection on oneself and the organization (i.e., awareness of one’s perception of one’s place in the work environment Römgens et al., 2020). In summary, career development is related to career identity, career self-management, reflection on oneself and the organization, and developmentally relevant employability capital (Figure 1).

### Employability dimensions of engineering students

The employability of engineering students is reflected in four dimensions, which are summarized as follows:

#### Human capital

Human capital refers both to job-specific and generic knowledge as well as meta-cognitive knowledge, skills and attitudes that enable individuals to thrive in the labor market (Rothwell et al., 2008; Römgens et al., 2020). Human capital is a core component of graduate employability that encompasses recognized competencies (e.g., problem-solving, critical thinking, or teamwork skills) and professional skills (Holmes, 2013; Clarke, 2018). Engineering students should systematically acquire the basic theoretical knowledge and professional skills necessary for the profession (Zhang et al., 2019). The knowledge level specifically refers to the mathematical, natural science, and engineering fundamentals as well as expertise that can be utilized to solve complex engineering problems; whereas the skills level includes skills that are specific to the engineering profession, such as design and development, application of modern tools, project management and financial analysis, research skills, and engineering analysis. With the rising trend of technology in, for instance, the construction industry, employers are seeking graduates who have the ability to use technology to effectively perform industry functions and therefore also need computer and technical skills (Arain, 2010). In addition, the skill level involves analytical skills, critical thinking and engineering thinking skills. However, experience in industry-sponsored projects or internships in industry or other workplaces also develops problem-solving skills that are important for engineering employability (Coll, 2015). In summary, in the human capital dimension, the employability of engineering students is specifically expressed in terms of engineering expertise and skills, engineering thinking skills, critical thinking and the ability to solve complex engineering problems, as well as relevant work/internship experience.

#### Social capital

Social capital has always been one of the dimensions elaborated in all classifications of employability. Social capital refers to the degree of embeddedness in relevant social networks and the knowledge, skills and attitudes to develop relevant working relationships (Römgens et al., 2020). Further, Fugate et al. (2004) define social capital as the *“goodwill inherent in social networks”* and *“the size and strength of a network.”* In addition to social networks, social class and university ranking are also considered as social-level employment influences, and graduates from higher social classes and higher-ranked universities are more likely to be employed upon graduation, as is the case for engineering majors (Peeters et al., 2019).Therefore, from the perspective of social capital, social class and university ranking, social network interactions are important factors influencing the employability of engineering students.

#### Individual attributes

Adaptability and flexibility are considered to be the two main components of individual attributes (Clarke, 2018). Fugate et al. (2004) defined personal adaptability as *“the ability to adapt to changing situations determined by personal characteristics that predispose individuals to engage in (pro)actively adaptive efforts, such as propensity to learn, locus of control and self-efficacy.”* Flexibility is an important dimension that underpins most employability frameworks, and it involves being proactive and reactive in developing one’s willingness and ability to adapt to changing situations and circumstances (Qenani et al., 2014; Römgens et al., 2020). For engineering students, the ability to engage in lifelong learning is one of the most emphasized competencies, which refers to the awareness of self-directed and lifelong learning as well as an ability to continuously learn and adapt to development, and it is a key activity to ensure that individuals are always at the forefront of the field (Stolk and Martello, 2015). Innovation ability is the ability to identify new problems and adopt new approaches to solve such problems through innovative thinking activities, and it is the fulcrum that guarantees learners the flexibility to respond to changes and actively develop themselves. Among the existing studies on engineering students’ employability, sociability and teamwork skills are highly valued and they are seen as lubricants for individual career development (Yusof et al., 2012). Therefore, adaptability and flexibility are reflected in engineering students’ personal traits, such as learning ability, sociability, teamwork ability, and engineering innovation ability.

#### Career development

**Figure 2:**
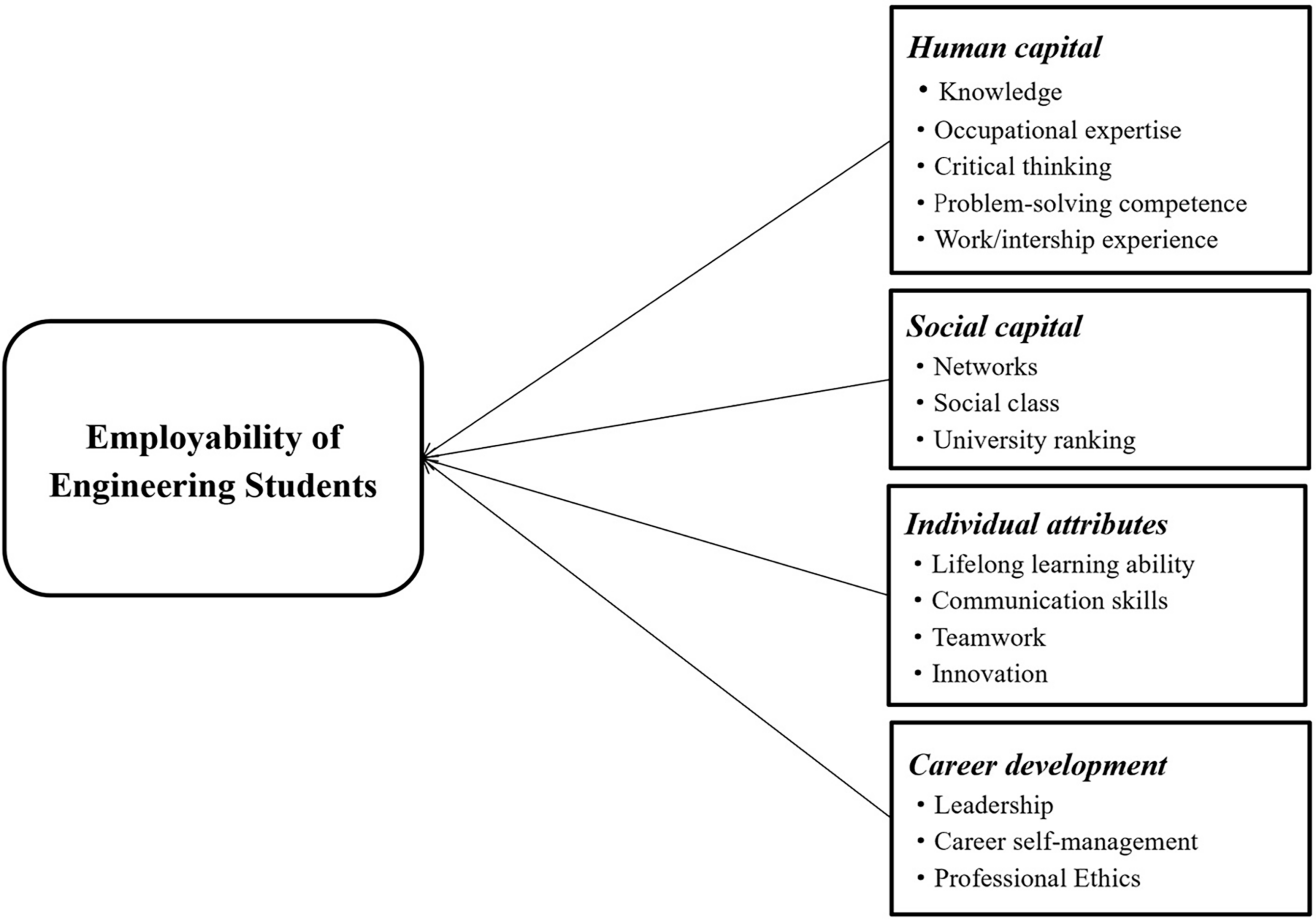
Core attributes of the employability of engineering students.

Career development refers to having a sense of career identity, whereas career self-management refers to the ability to manage and plan a career (Fugate and Kinicki, 2008; Römgens et al., 2020). Occupational identity is related to how people define themselves in an occupational context. This includes goals, personality traits, values, beliefs, and norms (Fugate et al., 2004). Occupational self-management refers to assessing one’s own abilities and personal awareness in terms of values, attitudes, competencies, interests, and work balance that can drive one’s career development. Reflection on one’s self and organization refers to the awareness of one’s position in the work environment, including awareness of personal goals, values, interests, expectations and motivations, as well as strengths and weaknesses (Römgens et al., 2020). Employers in the engineering field value the organizational leadership and self-management skills of employed individuals, and graduates with leadership traits demonstrate self-confidence, teamwork skills, and initiative (Aliu and Aigbavboa, 2020; Musa and Idris, 2020). In turn, self-management skills are key to helping employed individuals work effectively and complete tasks in a timely manner (Samavedham and Ragupathi, 2008). In addition, the US Accreditation Board for Engineering and Technology (ABET) and the Engineering Council of South Africa (ECSA) have listed professional ethics as one of the six core professional competencies required of engineering graduates. Engineering ethics is a reflection of social responsibility and is necessary to achieve a sustainable career (Smith et al., 2021). Based on this, the career development dimension of engineering students’ employability mainly includes personal qualities, such as organizational leadership, self-management ability, as well as engineering ethics and morality. In summary, the core attributes of the employability of engineering students can be seen in Figure 2.

### Current status and outline of the big data analytics courses in China and other countries

Big data analytics has been applied to many fields, such as engineering, medical and business areas, which is of great significance in the development of today’s technology and society. In order to meet the urgent needs of society for big data graduates of different majors, and improve the data analysis ability and data literacy of college students, many universities in different countries have offered big data analytics courses (Rao et al., 2019). Examples of universities in the United States currently offering big data analytics courses include Wright State University, Stetson University, Carnegie Mellon University, and Columbia University. In China, there are also universities offering big data courses, such as the University of Chinese Academy of Sciences, Harbin Institute of Technology, Xi’an Jiaotong University and Central South University (see Appendix 1). The courses tend to be concentrated in certain areas, such as computer science and engineering, information management, internet of things and software engineering, as well as general big data analysis and mining courses. In addition, big data analytics courses tend to be open to statistics and accounting as well as other majors.

#### Core curriculum and specific contents

Based on the course outlines of the big data analytics courses offered by the above eight universities, the core content of the courses are summarized in Table 2.

- All eight universities have an introduction to platforms and tools section that focuses on basic platforms such as Hadoop, Spark, and other tools related to big data; with the goal of ensuring that students are properly selecting data processing tools (Eckroth, 2018).
- Big data storage involves storing and managing data in a scalable manner that meets the needs of applications with the required access to the data (Strohbach et al., 2016). There are various data storage tools such as HBase, NoSQL, HDFS, and GFS, and these novel storage technologies offer improved scalability with lower operational complexity and cost (Gandomi and Haider, 2015). Big data also has the capability to store, manage, and analyze large amounts of heterogeneous data, which means it has the potential to spawn a data-driven society and economy with great transformative potential (Manyika, 2011).
- Data analysis, which is the core of this course, refers to a process of data analysis in which insights are gained by analyzing data and transforming useful information into knowledge relevant to big data (Ferraris et al., 2019). This can be considered as a sub-process within the overall process of extracting information from big data (Gandomi and Haider, 2015).
- Data visualization is an important step in presenting the results and insights gained through analysis and is critical in today’s data-driven world. It has been widely used to help people make decisions and is an important reference for modern business intelligence and successful data science applications (Qin et al., 2020).

**Table 2:** Topics of big data analytics course.

	A	B	C	D
Asamoah et al. (2017)	√	√	√	√
Eckroth (2018)	√		√	√
Introduction to Big Data Analytics Course in Carnegie Mellon University (n.d.)	√	√	√	
Big Data Analytics Course in Columbia University (n.d.)	√	√	√	√
Introduction to Big Data Analytics Course in University of Chinese Academy of sciences (n.d.)	√	√	√	√
Harbin Institute of Technology's Big data analytics course (n.d.)	√	√	√	√
Big Data Analytics Course at Xi'an Jiaotong university (n.d.)	√	√	√	√
Introduction to Big Data Analysis major in Central South University (n.d.)	√		√	√

A: Platforms and tools introduction; B: Data Storage; C: Data Analytics; D: Data Visualization.

#### Educational dimensions of the big data analytics course

According to the literature review, this section summarizes the educational objectives of the curriculum. In this study, all educational skills were divided into hard skills, which are those directly included by the curriculum, and soft skills, which are those that are gained indirectly.

##### Hard skills

###### Knowledge skills

Big data analytics courses can improve students’ learning efficiency and maximize their knowledge retention (Shabihi and Kim, 2021). Digital tools and platforms help students to search databases and websites for specialized information related to specific fields of study, thereby increasing their sources of knowledge acquisition (Moreira and Manuel, 2010). In this context, big data analytics courses have a positive impact on the knowledge level of engineering students.

###### Technology skills

Big data analytics courses include the learning of various technologies and tools, and all four universities mentioned previously conduct big data analytics courses that include an introduction to big data platforms and tools, such as Hadoop, HBase, MongoDB, and NoSQL (Saggi and Jain, 2018). Learning how to use big data platforms and tools can enrich students’ technical knowledge and improve their skills (Asamoah et al., 2017). Moreover, technical skills in big data analytics can be used to extract meaning from data, exercise insights, and provide integrated solutions for a variety of application domains (Iqbal et al., 2020). Therefore, big data related technology is also one of the main technical components of this course.

###### Analytical skills

The core purpose of a big data analytics course is to develop students’ data analysis skills (Introduction to Big Data Analytics Course in Carnegie Mellon University, n.d.; Big Data Analytics Course in Columbia University, n.d.; Asamoah et al., 2017; Eckroth, 2018). The goal of the course is to enable students to apply data mining principles to analyze large and complex data sets, master big data analytics methods and tools that transform data into valuable information. The course provides students with a systematic introduction to the theory and algorithms of big data analytics, including classical algorithms, such as primary data mining and advanced relational data mining as well as collaborative filtering. It also provides training on the application aspects of text big data analytics, knowledge computing, web data mining, and social media analysis. Learning big data analytics techniques improves students’ analytical skills and enables them to effectively use hardware and software knowledge, relevant engineering knowledge, and modeling methods to derive and analyze complex computer engineering problems, which helps improve the efficiency and reduce the cost of problem-solving (Ahmed et al., 2017). There is no doubt that analytical skills are an important educational goal of this course.

##### Soft skills

###### Decision-making competency

Access to big data provides opportunities to improve students’ competencies, including systematic thinking, collaboration, and problem-solving skills. Also, appropriate data drives the development of decision-making skills (Roy et al., 2017). Among the competencies, previous researchers mainly focused on decision-making. Indeed, big data analytics is increasingly becoming a trend that extracts massive amounts of data and information to support decision-making through the use of big data technologies, thereby enabling users to process the data more effectively and also helps in making relevant decisions (Saggi and Jain, 2018). In conclusion, digital technology and big data derived information can help to improve the decision-making ability of engineering students.

###### Critical and creative thinking

Asamoah et al. (2017) identified that a big data analytics course provided students with the ability to explore concepts beyond standard boundaries (Asamoah et al., 2017). Big data allows us to consider problems that have never been thought of or some problems that have not previously been solved (Roy et al., 2017). Furthermore, massive data and data analysis processes can enhance creative thinking and innovation (Kanth et al., 2018). Therefore, big data analytics courses can play a stimulating role in critical and creative thinking of students.

###### Communication skills

Communication skills are the ability to communicate and disseminate ideas and knowledge through any form of expression or technical medium (Moreira and Manuel, 2010). Digital training methods can improve students’ identification, problem-solving, visualization and communication skills (Lickteig, 2000). Moreover, Asamoah et al. (2017) emphasize that students should be able to effectively explain and communicate ideas through written and oral reports, and this ability can be trained through visualization and presentation sessions in big data analytics courses (Asamoah et al., 2017). The above literature sources highlight that students’ communication skills can be improved by learning how to use big data analytics.

**Figure 3:**
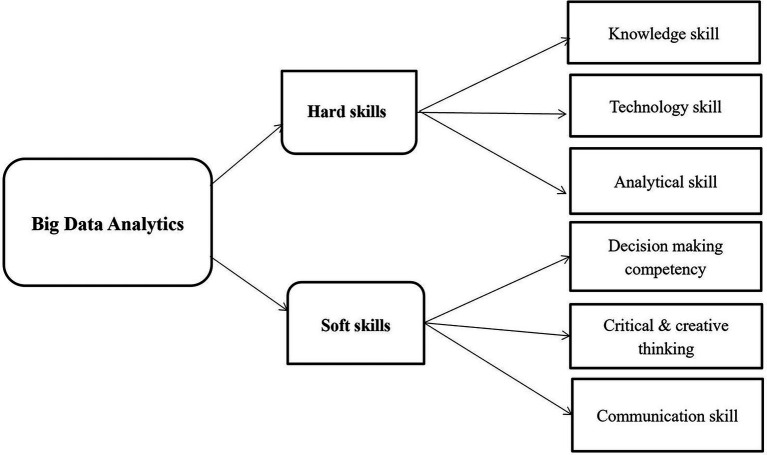
Educational dimensions of the big data analytics course.

According to the aforementioned review of the extant literature, the basic framework for the educational dimension of the Big Data Analysis course is constructed, as shown in Figure 3.

## Theoretical model and research hypotheses

**Figure 4:**
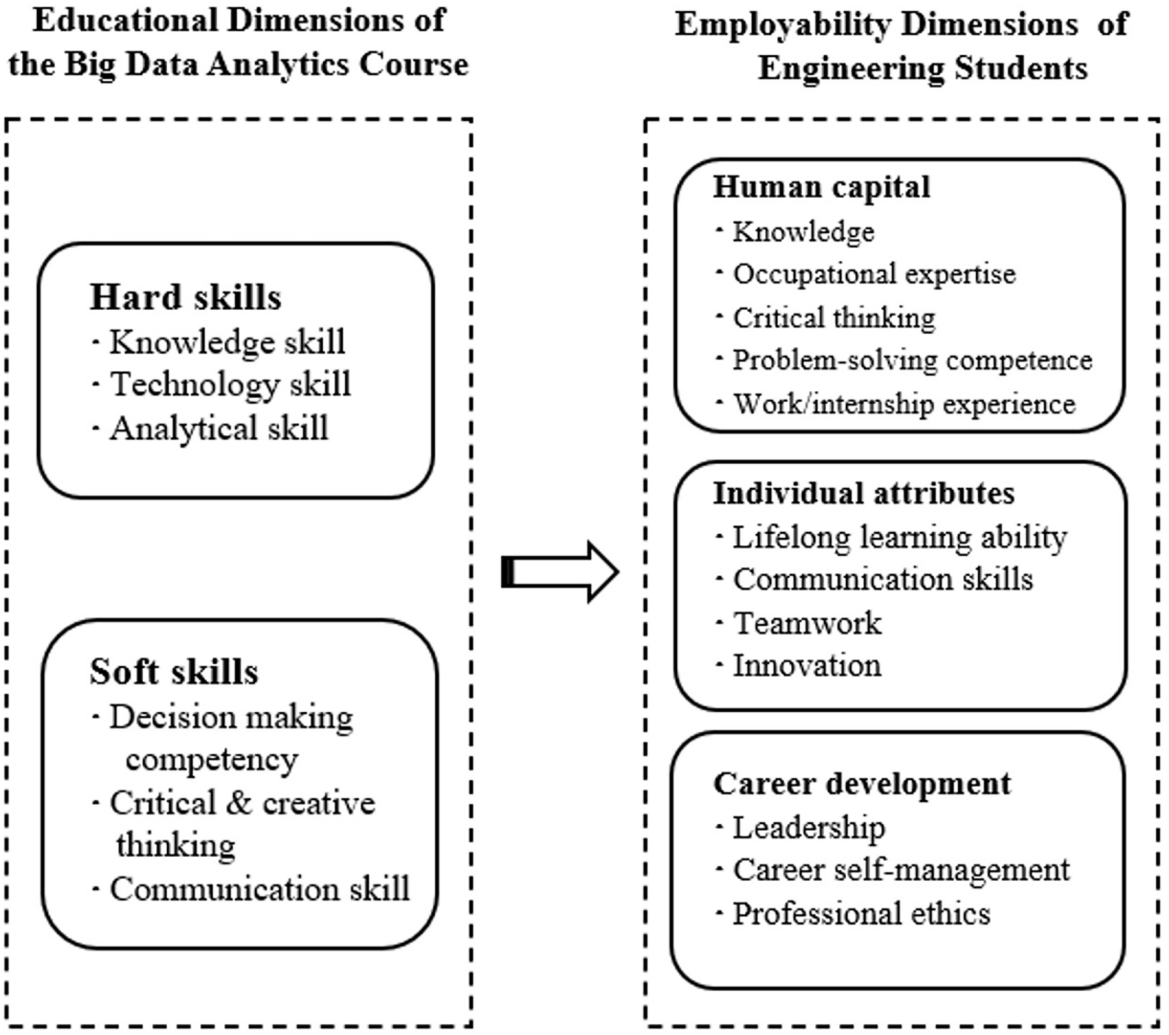
Educational dimensions of the BDA course and employability dimensions of engineering students.

Since the social capital dimension in employability focuses on the influencing factors at the social level, the educational dimension of big data analytics courses mainly focus on the level of personal ability. Therefore, this study focuses on the impact of this course on the three dimensions of human capital, individual attributes and career development in addition to social capital, as shown in Figure 4.

### The relationship between big data analytics courses and human capital of engineering students’ employability

The fundamental purpose of engineering education is to educate students to become professional engineers. This requires students to have a thorough and specific understanding of scientific and engineering principles, as well as a general understanding of the complex products, processes and systems that make up today’s society and industrial structures (Nicolescu and Nicolescu, 2019). Students can enhance the development of higher-order thinking skills through an understanding of professional practice, applying formal knowledge and gaining relevant generic skills to practice decision-making (Coff, 2002). Meanwhile, students need to make progress in their ability to analyze cases, solve critical problems, and think critically. After reaching a sufficient level of expertise, students use their knowledge to solve more complex problems (Ramberg et al., 2019). In this context, critical thinking is widely recognized as an important skill, and the ability to apply knowledge is a key component of critical thinking (Douglas, 2012). Critical thinking relies on a thorough knowledge and understanding of the content and epistemology of the discipline and cannot be divorced from a specific subject area (Yu et al., 2015). For instance, it is difficult for a person who knows little about nuclear physics to become a critical thinker in the field of nuclear physics, so a broad and deep knowledge of the discipline is essential for critical thinking in the field.

The core competencies of big data technologies include mastery of big data platforms (such as Hadoop, Spark, and Hive), programming languages (such as Java, and Python), and databases (such as SQL and NoSQL), and encompass a wide range of knowledge areas and skills. This means that engineering professionals who master big data technologies can take on different roles and responsibilities in different big data workflows (Gurcan, 2019). Thus, big data related technologies improve engineering students’ professional skills, such as design and development, and also improve their ability to explore different engineering applications in the workplace. The development of distributed computing and the advent of high-speed computers have made data processing much faster and cheaper, for example, the Hadoop platform enables decentralized clusters of machines to run cooperatively to achieve improved performance (Mauro et al., 2017).

Big data analytics courses improve engineering students’ data analysis skills and equip them to work as business analysts or data analysts (Gurcan, 2019), which can enable students to think more critically when facing problems at work and be able to solve a series of workplace problems more comfortably. In business, the job responsibilities of a business analyst are firstly, to drive the company’s decision-making by analyzing large amounts of data to influence marketing strategies and increase sales; and secondly to provide analytical support for the company’s business plan and report on relevant strategic ideas (Mauro et al., 2017). In this process, business analysts gain expanded knowledge, enhanced professional competencies, and improved critical thinking and problem-solving skills through the process of analyzing company data. Students therefore need to use critical thinking skills to identify what they need to do in their work and how to do it, and critical thinking skills are developed through the process of examining big data through immediate data analysis (McBride and Philippou, 2022).

In summary, we propose the following research hypothesis regarding the hard skills in big data analytics courses to enhance the human capital dimension of engineering students.

*H1a:* The hard skills (such as knowledge, technical and analytical skills) developed in big data analytics courses have a significant positive impact on the human capital of engineering students.

A decision is a choice or judgment that an individual or organization has to make about something. An understanding of decision-making can provide students with the tools and intellectual background necessary to develop higher-order thinking processes that enhance higher level learning skills, such as analysis, synthesis, and evaluation (Alkhatib, 2019). Moreover, the development of decision-making skills can positively enhance students’ thinking patterns and learning habits, and provide a more comprehensive and solid grasp of engineering knowledge and analytical skills (Samavedham and Ragupathi, 2012). In addition, decision-making skills are complemented by critical thinking skills, which is the ability to have a deep understanding of things and be able to analyze problems rationally and thus make appropriate decisions more effectively. This is especially important for engineering professionals who need to make important decisions regarding engineering systems and programs (Ahern et al., 2019). Therefore, engineering students with good decision-making skills are able to conduct critical analysis when solving problems, think about the effectiveness of solutions, and finally make correct decisions on the engineering problem.

Critical thinking skills are associated with a range of positive outcomes, including improved problem-solving and information processing skills, as well as reduced irrationality, subjectivity, and bias. The development of critical thinking in engineering students can be fostered through analysis of data combined with extensive practice. Critical thinking is highly desirable in the workplace, where engineers must think logically to identify, synthesize, analyze, and solve problems in order to properly apply it to streamline work processes (Grecu and Deneş, 2020). In a recent survey, engineering employers placed the importance of “*the ability to think independently and critically*” ahead of logical and sequential thinking and academic learning skills (Nelson, 2001). In addition, modern engineers should have strong expertise and creative problem-solving skills to deal with uncertainty and new situations in the workplace, and provide solutions to urgent technical cases (Zhang et al., 2019).Therefore, critical thinking is essential to improve the ability to solve engineering problems creatively.

Many employers are now looking for graduates with strong communication skills. Formal communication skills, such as writing and presentation skills, are essential for career development and an asset for engineers in industry and other workplaces (Bojovic et al., 2015). In the workplace, engineering graduates sometimes become leaders and supervisors who need to know how to give appropriate instructions (Masduki and Zakaria, 2020). Thus oral communication skills need to be applied in realistic work scenarios, which are helpful for engineers to solve practical problems. In addition, it has also been documented that communicative competence partially mediates students’ ability to improve their problem-solving skills.

Therefore, we propose the following research hypothesis for soft skills developed by the big data analytics course on human capital development for the employability of engineering personnel:

*H1b:* Soft skills (such as decision-making, critical thinking and communicative skills) developed in big data analytics courses have a positive impact on human capitaland the subsequent employability of engineering students.

### The relationship between the big data analytics course and the individual attributes of engineering students’ employability

Professional knowledge as well as student attitudes and the learning environment are all crucial to promote the lifelong learning skills of engineering students. Indeed, it has been identified that the more educated and knowledgeable people are, the more they possess the skills for lifelong learning (Guest, 2006). In the ever-changing development of engineering technology, people need to be willing to keep learning new things and technologies, and develop themselves continuously using advanced information and networks. This form of continuous professional development is considered a key component of lifelong learning ability (Genco et al., 2012). Moreover, these individuals tend to possess high levels of creativity and often have a wide range of creative outputs in their learning and work. All of this contributes to personal and professional development.

In modern society, educational institutions train students not only to acquire knowledge and skills, but also to guide them to adapt to future social changes and avoid being disadvantaged by the ever-changing development, so that everyone can benefit from having the attitude and awareness of continuous learning throughout their lives (Zhang et al., 2017). Furthermore, international organizations and domestic experts have successively proposed the components of lifelong learning competencies. Such components in descending order of priority are as follows: communication competencies, problem-solving competencies, cooperation competencies, information competencies, data competencies, technology applications, autonomous development, language competencies, responsibility, culture, and innovation awareness and creativity (Murumba and Micheni, 2017). Among them, information competency, data competency and technology application can be improved from learning the big data analytics course, while allowing engineering students to grasp the emerging concepts of big data analytics technology in the era of Industry 4.0. This is conducive to their continuous discovery and helps engineering students to develop good awareness of emerging technologies as well as abilities in lifelong learning (Murumba and Micheni, 2017). In addition, research by Pham et al. (2021) determined that big data technology can enhance students’ innovation ability through design thinking (Pham et al., 2021).

Big data analytical capabilities can be divided into four dimensions at the enterprise level, which are technical capabilities, management capabilities, personnel capabilities, and predictive capabilities (Akter et al., 2016). Furthermore, big data analytical and predictive capabilities can be viewed as the most essential capabilities that provide a rich source of information for enterprises and individual employees to organize effective learning and analytic activities (Yang and Wang, 2020). This has a positive effect on individual organizational learning capabilities as well as a positive effect on technological innovation. Also, the literature has verified that big data analytical and predictive capabilities have a significant effect on the improvement of individual innovation capabilities (Zhang et al., 2019). A study from 2019 further stated that skills on big data analytics can have a direct positive effect on product innovation capabilities, process innovation capabilities, management innovation capabilities, and marketing innovation capabilities. In addition, a study by Niebel et al. (2018) expressed a similar view that big data analytics skills can contribute to innovation capabilities (Niebel et al., 2018).

Based on the reported literature sources, the following research hypothesis is proposed regarding the hard skills in big data analytics courses to enhance the individual attributes dimension of engineering talents.

*H2a:* Hard skills (such as knowledge, technical and analytical skills) developed in courses on big data analytics have a positive effect on the development of individual attributes of engineering students.

Today’s complex social, economic, engineering, and scientific activities often require effective teamwork tosecure successful outcomes. Teams are usually composed of members with different competencies, and those with stronger decision-making skills have an important role in the team (Yu et al., 2017). As a core member of the team, their ability to cooperate and communicate while playing a leading role in the team in problem-solving increases dramatically. Moreover, it was determined that the inclusion of decision-making skills also has a positive effect on the adaptability and flexibility of engineering students’ employability (Munir, 2022). To cope with uncertainty and change, adaptability and flexibility are also considered as key attributes of engineers’ soft skills and this implies the ability to change and adapt to a range of conditions that are essential for competitiveness.

Engineering students who are critical and creative thinkers tend to have a deeper understanding of things as well as unique insights, and are generally more likely to generate creative ideas and innovative ways of solving problems through comprehensive and logical analysis (Shoop and Ressler, 2011). Indeed, studies have shown that engineering students are able to develop a deeper understanding of issues and problems when conducting group experiments, exploratory discussions and brainstorming on practical problems, as well as collaborative forms of working. More importantly, the ability to critically approach their hypotheses and inferences, as well as communicate and argue in a way becomes an important outcome (Ahern et al., 2019). Critical thinking is the key to communication, which undoubtedly strengthens team cohesion and communication skills while stimulating the development of critical thinking. In addition, engineering students with this competency tend to plan their careers systematically, and actively ensure that they become lifelong learners, which can serve as a guide to achieve their career and life goals (Martinez-Mediano and Lord, 2012).

Interpersonal communication skills include cooperation, tolerance and listening, which are critical for teamwork and can help an individual avoid misunderstandings and conflicts at work (Masduki and Zakaria, 2020). Furthermore, active listening is crucial because it is a tool for understanding and interpreting verbal as well as nonverbal communication (Lenard and Pintarić, 2018). In engineering situations, a teamwork project usually includes important communication tasks, such as project design reports, project progress reports, and project presentations. Therefore, effective teamwork requires that engineers are able to communicate effectively in both formal and informal interpersonal interactions (Bojovic et al., 2015). According to the perspective of a analytics course (which includes material on data visualization and presentation), engineering studentsbecome equipped with certain communicative skills that help them to better integrate themselves intoteam projects and complete their corresponding tasks.

In summary, we propose the following hypothesis based on the findings from the extant literature.

*H2b:* Soft skills (such as decision-making skills, critical thinking and communication skills) developed in big data analytics courses have a positive effect on the development of individual attributes of engineering students.

### Influence relationship between the big data analytics course and career development of engineering students’ employability

Highly educated employees tend to develop and maintain influential social networks that create value throughout their careers. Indeed, knowledge workers with a high level of professional skills are key to engineering-related business performance (Shah and Nowocin, 2015). They could potentially be the primary provider of an organization’s core services, or ensure effective business support services (Okolie et al., 2020). This capability is often the hardest to replicate, which can take the longest to develop, and is often extremely scarce in the labor market (Majid et al., 2019). Surveys have found that graduates with more comprehensive engineering expertise and skills generally have higher salaries than others and are more attractive to employers. Possessing professional skills also has a certain positive guiding effect on the improvement of technical leadership in the workplace, because leaders and managers need rich knowledge reserves combined with comprehensive skills (Hirsh, 2006). In conclusion, the acquisition of a broad range of knowledge with a variety of professional skills has been generally recognized to facilitate effective career management and development, andimprove individual as well as organizational outcomes (Fox-Turnbull et al., 2022).

Technology is a necessary prerequisite for occupational survival and development. Combining the principles and practice of big data technology to educate students will lead to the big data analytics and processing course being more suitable for engineering students’ personal career development (Gao et al., 2018). Engineering students who have mastered big data technology, combined with their own engineering knowledge and skills, will have an improved understanding of what they can do and their value. This will allow engineering students to think hard about their life goals and connect career aspirations with personal growth and values (Snieder and Zhu, 2020).

Under the social background of the world’s unprecedented level of changes, the economic, life and employment situation is often complicated and unstable. When facing the career choice, engineering students can analyze the massive amount of information on the internet to obtain effective data that is promising and beneficial to their career planning, which helps them choose the right career (Tang and Meng, 2021). This is the improvement of self-management ability. At the same time, big data analytics skills are a popular application nowadays, and educational institutions offer big data analytics courses according to this development trend. Thereby keeping pace with the times and giving engineering students more employment options, which is conducive to the cultivation of students’ self-management skills (Zhang et al., 2017). Furthermore, research by Osipov et al. (2021) shows that as an individual grows, he or she will understand that taking responsibility for oneself is an important ability, since no one can always walk through every step of life smoothly (Osipov et al., 2022). The improvement of the analytical ability of engineering students will potentially make them realize this earlier and improve their career self-management ability. Consequently improving analytical skills can help engineering talent advance in their careers (Chandhok, 2021).

Therefore, this study proposes the following research hypothesis in regard to how the development of hard skills on big data analytics course improves the dimension of career development of engineering students:

*H3a:* Hard skills (such as knowledge, technology and analytical skills) developed in big data analytics courses have a positive impact on the career prospects of engineering students.

Highly educated employees tend to have abstract experiences, strong decision-making abilities, and career planning skills, which have a positive impact on their careers. Decision-making ability is a basic personal attribute that can affect the success of career development (Xu et al., 2022). That is, the extent to which a person has acquired the skills required for effective decision-making that may facilitate positive outcomes for individuals and organizations in career management, including the achievement of sustainable career paths (Ceschi et al., 2017). In addition, in today’s rapid technological development, some routine jobs are increasingly replaced more and more by robots, and people with effective decision-making ability become competitive in the job market (Kenayathulla et al., 2019). This is because they are able to make open-ended decisions in the event of an emergency, which also explains why employers have always used decision-making competency as one of the most basic needs for recruiting employees.

Career management is the process of developing career goals and strategies, and obtaining feedback on career progress through insights into oneself and the environment. Students with critical thinking have the awareness of thinking about career development in a timely manner, and will make reasonable plans for their careers (Seema and Sujatha, 2013). Critical thinking by engineering students enables them to identify strengths and weaknesses in their own thinking and know how to improve as well as thinking holistically on engineering ethics (Nicolescu and Nicolescu, 2019). They are generally willing to ‘jump out of the comfort zone’ and try challenging new activties thereby becoming more highly skilled with good problem-solving, decision-making, and communication. In this case, students are able to realize that leadership has a positive impact on employability as well as future career development prospects.

The ability of graduates to communicate effectively can have a significant impact on their career development (Masduki and Zakaria, 2020). Research by Otto et al. (2019) ascertains that communication skills are associated with objective career success, including salary and position (Otto et al., 2019). The improvement of communication skills can promote engineering students to actively participate in extracurricular activities organized by the academic institution or the student union. Participating in these activities can attract students and peers with common interests to communicate and learn from each other, gain self-confidence, and secure decision-making ability as well as team-building ability through social interactions, thereby improving their own leadership ability (Osipov et al., 2021).

In view of these findings from the literature review, the following research hypothesis is proposed:

*H3b:* The soft skills (such as decision-making ability, critical thinking, and communication skills) developed in the big data analytics course have a positive impact on the career prospects of engineering students.

**Figure 5:**
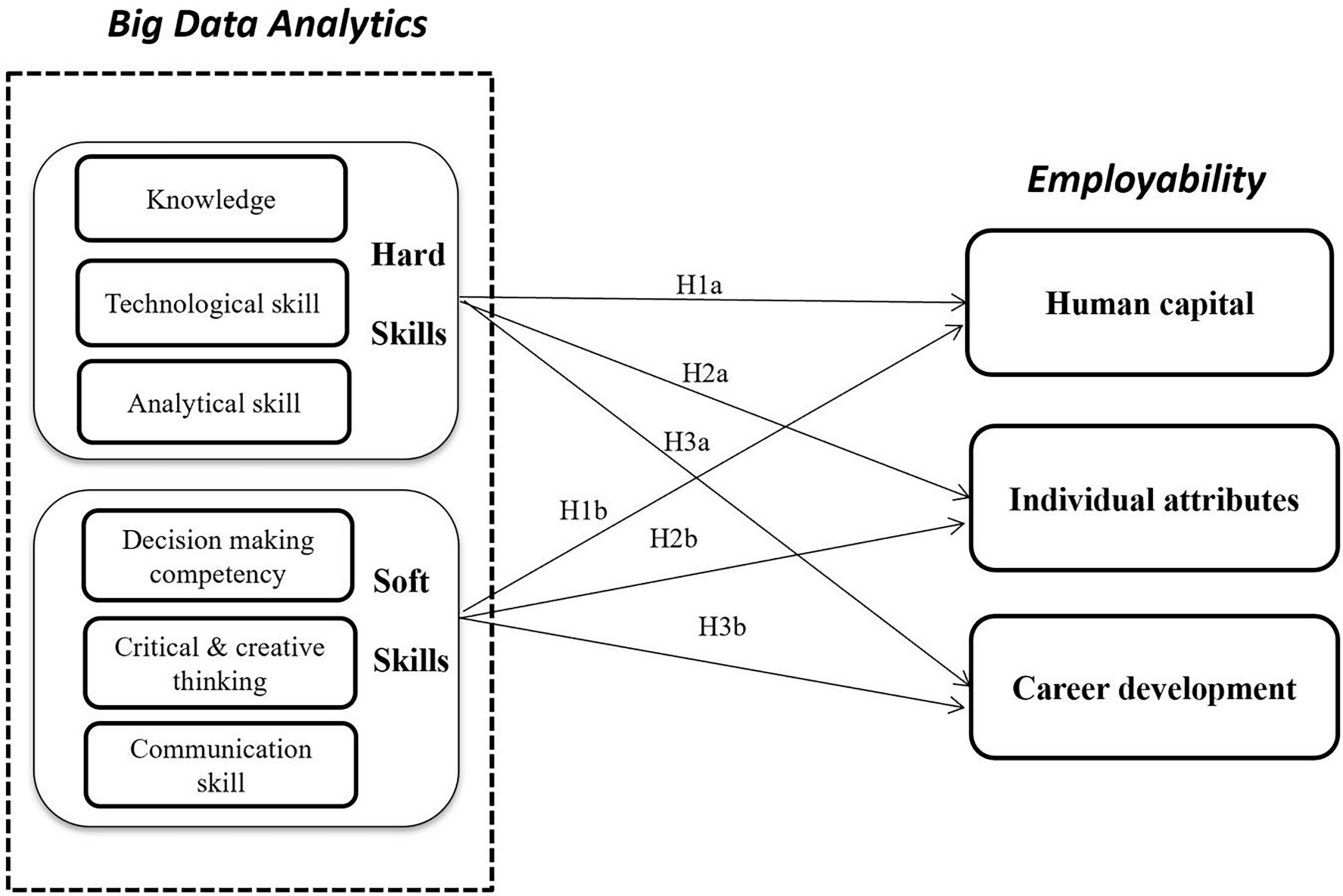
Conceptual model of BDA-E impact mechanism.

Based on the above analysis, the conceptual model for employability supported through adoption of the big data analytics course (BDA-E) is constructed in this study, as shown in Figure 5.

## Materials and methods

### Data collection and participants

In this empirical study, data were collected by means of a questionnaire survey. In order to further understand the influence of digital training on the employability of engineering students and ensure the reliability and validity of the measurement items, this study is based on the findings of existing literature studies as well as selected mature subscales. The measurement items of variables are designed according to the principles of simplicity and accuracy for the situation of engineering majors and combined with specific research questions.

The survey was designed and conducted between March 30 and October 15, 2021 using Sojump to distribute questionnaires to engineering students of different majors, 350 questionnaires were distributed and 213 questionnaires were collected. After matching and sorting, excluding invalid questionnaires, such as removing invalid questionnaires with more consistent answers to question items, or contradictory answers before and after, 155 valid matching sample data of questionnaires were finally obtained, with a valid response rate of 72.7%. The questionnaires were answered anonymously, and participants were informed of the purpose and importance of the survey, as well as the results of the questionnaires were only used for academic research, and the privacy and security of the participants were guaranteed to be effectively safeguarded, so as to receive as many true and valid responses as possible. Information was investigated using SPSS24.0 and structural equation modeling (SEM) in MPLUS.

As can be seen from Table 3, 38.71% of the students who participated in the questionnaire were male and 61.29% were female; the mean age of these students was 21.43 years (SD = 1.65); 10.32% had a master’s degree or above, and 89.68% had a bachelor’s degree, which reflected that the participants in this survey had a high level of education in general.

**Table 3:** Profile of participants.

Demographic variable	All (*N* = 155)	
	Frequency (%)	
*Gender*		
Male	60	38.71
Female	95	61.29
*Age* (*M* = 21.43 SD = 1.65)		
18–25	150	96.77
26–30	3	1.94
31–40	2	1.29
*Education*		
Bachelors	139	89.68
Masters	15	9.67
*Region*		
Beijing	39	25.16
Xi’an	48	30.97
Shenzhen	30	19.35
Changsha	38	24.52

### Measurement

The questionnaire contains three parts, the first part mainly investigates the demographic information; the second part mainly investigates the impact of big data analytics courses on engineering students’ knowledge skills, big data analytics skill, technical ability, communication ability, decision-making ability and critical thinking ability, with 28 questions in total. The third section investigates the self-perceived employability of engineering students, including knowledge skills, lifelong learning skills, problem-solving skills, teamwork skills, innovation skills, and leadership skills, with a total of 28 questions (see Appendix 2). All questions were scored on a 5-point Likert scale, where 1 means “strongly disagree” and 5 means “strongly agree.”

### Data analysis

#### Treatment of data

To ensure the quality of the data before starting the data analysis, all completed questionnaires (*N* = 155) were checked for completeness and more than 5% of missing items, as recommended by Seo (2005). All questionnaires were answered completely. Each item was coded as favorable or unfavorable (Seo, 2005). For items scoring <3, the item represents unfavorable, otherwise, it means favorable.

#### Statistical analysis

Structural equation modeling (SEM) program was used to analyze the data, and MPLUS software was used to construct SEM analysis of BDA-E influence mechanism. Different types of overall fit indices were used to evaluate the SEM models in this study, including *χ*^2^/degrees of freedom ratio (*χ*^2^/df), Comparative Fit Index (CFI), Tucker-Lewis Index (TLI), and Root Mean Square Error Approximation (RMSEA).

##### Absolute fit indexes

The absolute fit index is often used to determine the “poor fit,” where *χ*^2^/df is the commonly used absolute fit index. If the ratio of *χ*^2^/df is <3, the model is considered acceptable (Wheaton et al., 1977; Marsh and Balla, 1994).

##### Incremental fit indexes

Incremental fit indices typically assess “goodness of fit,” with larger values indicating a better fit between the hypothesized model and the data. Commonly used incremental fit indices include Bentler and Bonett’s Normative Fit Index (NFI), Comparative Fit Index (CFI), Tucker-Lewis Index (TLI), and Incremental Fit Index (IFI), of which both CFI and TLI are used in this study. A CFI value >0.95 (ranging from 0.00 to 1.00) represents a good fit model. A TLI value >0.9 indicates a good fit (Bentler and Bonett, 1980; Lt and Bentler, 1999).

## Results

### Descriptive analysis and correlation analysis

The correlations between all dimensions are shown in Appendix 3, highlighting that all variables are significantly correlated (*p* < 0.05) and none of the correlation values exceed the threshold of 0.9, which indicates that there is no multicollinearity between items (Hair, 2006).

### EFA

The reliability of the variables can be tested by Cronbach’s alpha coefficient, if alpha >0.6, it indicates the validity of the scale, and the larger the alpha reliability coefficient indicates the better the reliability of the scale. The results of the reliability test using SPSS software are shown in Appendix 4. It can be seen that the alpha values of all variables are >0.7 and the Corrected Item-Total Correlation (CITC) values of all measures are >0.3, thereby indicating that the data of each variable meet the requirements of reliability.

Then, the validity of the factors was tested by exploratory factor analysis (EFA). The results of EFA are shown in Table 4. From Appendix 5, it can be seen that the Kaiser-Meyer-Olkin (KMO) values of the study variables are >0.7 and the Bartlett’s spherical test significance is <0.001, which is statistically significant. Meanwhile, Table 4 shows that the factor loadings of each question item are >0.6, the combined reliability is >0.8, and the mean variance extracted is >0.5. It can be observed that each question item belongs to the corresponding variable or dimension and has good discriminant validity and convergent validity that can be tested by SEM.

**Table 4:** The results of factor analysis.

Construct	Items	Factor loading	C.R.	AVE
Knowledge skill	KS1	0.885	0.924	0.801
	KS2	0.943		
	KS3	0.855		
Decision-making skill	DMS1	0.623	0.834	0.504
	DMS2	0.677		
	DMS3	0.857		
	DMS4	0.731		
	DMS5	0.636		
Critical thinking skill	CTS1	0.835	0.917	0.649
	CTS2	0.818		
	CTS3	0.724		
	CTS4	0.814		
	CTS5	0.827		
	CTS6	0.811		
Technology skill	TS1	0.902	0.947	0.856
	TS2	0.917		
	TS3	0.956		
Big data analytics skill	BDAS1	0.849	0.950	0.792
	BDAS2	0.913		
	BDAS3	0.907		
	BDAS4	0.887		
	BDAS5	0.893		
Communication skill	CS1	0.727	0.887	0.567
	CS2	0.754		
	CS3	0.677		
	CS4	0.744		
	CS5	0.817		
	CS6	0.795		
Human capital	KC1	0.834	0.937	0.650
	KC2	0.865		
	KC3	0.754		
	PSC1	0.767		
	PSC2	0.830		
	PSC3	0.845		
	PSC4	0.837		
	PSC5	0.705		
Individual attributes	LL1	0.827	0.956	0.598
	LL2	0.895		
	LL3	0.836		
	LL4	0.538		
	LL5	0.704		
	TC1	0.849		
	TC2	0.851		
	TC3	0.705		
	TC4	0.637		
	TC5	0.581		
	IC1	0.845		
	IC2	0.882		
	IC3	0.851		
	IC4	0.672		
	IC5	0.807		
Career development	LC1	0.815	0.896	0.634
	LC2	0.772		
	LC3	0.726		
	LC4	0.831		
	LC5	0.833		

C.R., Construct Reliability; AVE, Average Variance Extracted.

#### Hypothesis testing

**Figure 6:**
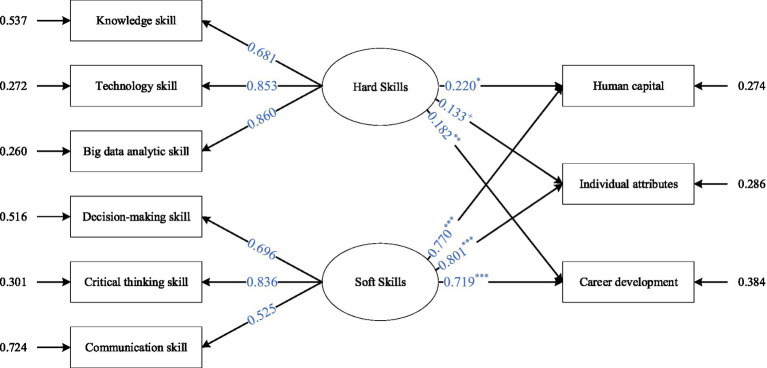
Overall results from the SEM. *χ*^2^/df = 2.968, CFI = 0.952, TLI = 0.913.

The hypotheses were examined using MPLUS through Bootstrap method and the results were represented in Figure 6. The specific coefficient results are shown in Table 5. The fit index of the structural equation model results was within the acceptable range, with *χ*^2^/df = 2.968 < 3, CFI = 0.952 > 0.90, and TLI = 0.913 > 0.90. It can be seen that the relationship model of the big data analytics course constructed in this study on the employability of engineering talents has a good fit.

**Table 5:** SEM results including coefficients.

Path	Estimate	SE	95% Bias-corrected CI	Hypotheses
Hard skill → Human Capital	0.220^*^	0.093	[0.048; 0.411]	Supported
Hard skill → Individual attributes	0.133	0.071	[−0.032; 0.254]	Not supported
Hard skill → Career development	0.182^**^	0.065	[0.046; 0.301]	Supported
Soft skill → Human Capital	0.770^***^	0.076	[0.593; 0.891]	Supported
Soft skill → Individual attributes	0.801^***^	0.062	[0.671; 0.910]	Supported
Soft skill → Career development	0.719^***^	0.078	[0.545; 0.849]	Supported

Bootstrap = 2000; SE = standard error; 95% Bias-corrected CI = 95% Bias-corrected confidence interval. ^*^p < 0.05, ^**^p < 0.01, ^***^p < 0.001.

Combining the SEM results in Figure 6; Table 5, it is determined that the variables of knowledge, technology skill and big data analytical skill are under the dimension of hard skills; and the classification and dimensions of variables of decision-making, critical thinking and communications skill are under the dimension of soft skills. The settings correspond exactly. Based on the data in Figure 6; Table 5, it can be observed that:

1. Hard skills have a positive effect on the human capital (Estimate = 0.220, *p* < 0.10) and H1a is verified. Bootstrapping BC 95% CI is (0.048, 0.411), excluding zero value, further supporting the positive effect of hard skills on the human capital dimension.
2. Soft skills have a significant positive effect on the human capital (Estimate = 0.770, *p* < 0.001) and H1b is verified. Bootstrapping BC 95% CI is (0.671, 0.910), excluding the zero value, further supporting the positive effect of soft skills on the human capital dimension.
3. Hard skills did not have a significant effect on individual attributes (Estimate = 0.133, *p*<). Bootstrapping BC 95% CI is (−0.032; 0.254), including the zero value, demonstrating that hard skills had no significant effect on Individual attributes.
4. There is a significant positive effect relationship between soft skills and Individual attributes (Estimate = 0.801, *p* < 0.001) and H2b is verified. Bootstrapping BC 95% CI is (0.671, 0.910), excluding the zero value, further supporting the significant positive effect of soft skills on Individual attributes.
5. Hard skills have a positive effect on career development (Estimate = 0.182, *p* < 0.01) and H3a is verified. Bootstrapping BC 95% CI is (0.046, 0.301), excluding the zero value, further supports the positive effect of hard skills on career development.
6. There is a significant positive effect of soft skills and career development (Estimate = 0.719, *p* < 0.001) and H3b is verified. Bootstrapping BC 95% CI is (0.545, 0.849), excluding the zero value, further supporting the positive effect of soft skills on career development.

Therefore, and on the whole, the soft skills cultivated by the big data analytics course have a greater impact on the employability of engineering majors than the hard skills.

## Discussion

From the empirical analysis, it can be concluded that the big data analytics course has significantly improved the engineering students’ abilities in two educational dimensions, namely soft skills and hard skills. The effects of the dimensions are all positively correlated. In addition, the results also found that soft skills have a more significant impact on the employability of engineering students than hard skills. The results underscore the positive impact and importance of digital training on the employability of engineering talents.

Firstly, we assume that the two skills dimensions of soft skills (including decision-making skills, critical and creative thinking skills, and communication skills) and hard skills (including knowledge skills, technical skills, and analytical skills) are both developed in big data analytics courses. It is further assumed that the skills have a positive impact on the human capital dimension of engineering students’ employability. The findings confirm this view, and we find that both skill dimensions of engineering students educated on big data analytics courses have a positive impact on human capital in the employability dimension. This result is consistent with the research of scholars such as Mauro et al. (2017), Masduki and Zakaria (2020), and Escolà-Gascón and Gallifa (2022), which indicates that among the three abilities of the hard skills dimension developed by the big data analytics course, the higher the knowledge level, technical skills and analysis skills, the higher the employability of engineering students (Mauro et al., 2017; Escolà-Gascón and Gallifa, 2022). Among the three soft skills cultivated in the course, the cultivation of decision-making ability and communication ability enhances students’ inherent thinking mode and learning habits, improves their learning ability, and correspondingly enriches students’ internship experience and ability to make systematic career planning (Masduki and Zakaria, 2020). It is also worth noting that the improvement of critical and creative thinking has the greatest impact on students’ human capital ability. Furthermore, we found that the stronger the students’ critical thinking ability in the data analysis course, the higher their level of engagement in various knowledge areas, and the more outstanding the problem-solving ability, which is a key requirement to be successful in the workplace.

Secondly, in the dimension of the individual attributes of engineering students’ employability, only soft skills have a significant positive impact. The results are consistent with the research findings of Grecu and Deneş (2020) and Yong and Ling (2022), which shows that the developing of critical and innovative thinking of students in big data analytics courses can also improve the innovation skill and lifelong learning ability of students. Further, the decision-making skills can improve the communication and team cooperation ability of engineering students (Grecu and Deneş, 2020; Yong and Ling, 2022). For example, engineering students who think critically and creatively have a comprehensive and logical analysis of problems and are more likely to generate creative ideas and innovative solutions to problems (Shoop and Ressler, 2011; Zhang et al., 2019). These students tend to systematically plan their careers, and actively ensure they become lifelong learners, which can serve as a guide to achieve their career and life goals.

Finally, the career development dimension in engineering students’ employability is also positively influenced by the two skill dimensions developed by the big data analytics course, which confirms previous studies of Munir (2022) and Fox-Turnbull et al. (2022). However, from the overall research results, soft skills have a greater impact on the employability of engineering students. In regard to campus recruitment, non-technical skills are indispensable to ensure the employability of engineering graduates, and the improvement of engineering students in soft skills is important to ensure the employability of graduates. Therefore, it is necessary to carry out digital training for engineering students, not only to improve the ability of students in the dimension of hard skills, but also to strengthen the cultivation of students’ soft skills.

## Implications

This study has practical implications for higher education institutions and engineering students, and makes theoretical and practical contributions to engineering education. The theoretical contribution is that the study broadens the development path of engineering graduates and employability prospects by exploring the influence and importance of big data analytics courses. The practical contribution is that this study strengthens the relationship between engineering graduate development and digitalization; promotes digital skills development for engineering students; and supports enhanced employability for engineering graduates. For engineering students, this study helps identify their shortcomings in employability and makes them better able to meet the requirements of employers in the job market.

## Conclusion

This study takes the big data analytics course as the starting point and studies the impact and importance of the employability of engineering graduates. The human capital, individual attributes and career development employability dimensions of engineering students are summarized through a comprehensive literature review. Additionally, the two educational dimensions of soft and hard skills developed by the big data analytics course are evaluated. Thereafter, a hypothesis model of the influence mechanism of BDA-E was constructed and a questionnaire designed on this basis, where structural equation model (SEM) was used as the test method. The results identify that the big data analytics course can improve both the soft skills and hard skills of engineering students. Moreover, soft skills have a more significant impact on the employability of engineering graduates.

Based on the results and implications of the study, the following suggestions are provided on how to improve the employability of engineering graduates.

1. Engineering educators need to change their teaching mode and improve their comprehensive teaching abilities. To develop big data analysis skills of students with comprehensive literacy, educators should first improve their own data analysis skills and various abilities, and clarify the needs of employers for engineering graduates in the era of big data. There is a further need to avoid structured and memory-based teaching method and make good use of digital training mode so as to improve the soft and hard skills of engineering students. This is especially the case to strengthen the training of students’ soft skills and help them prepare for employment.
2. Engineering educators should increase the number of big data analytics courses for engineering majors. It is important for engineering students to master the analytical consciousness and various abilities in the era of big data. Big data analytics courses should be carried out in the first year of higher education to make full use of its important role in providing comprehensive skills and enhancing the employability of engineering graduates. This will also improve the data analysis skills and problem-solving skills of students, and support soft skills development, such as communication, cooperation, leadership and innovation in team learning.
3. There is a need to build a big data analytics practice platform or enterprise cooperation to exercise the practical ability of students. Furthermore and through harnessing industry-university cooperation, students will be able to solve practical problems with the knowledge and technology they learn in the course and enhance their practical application ability, thus laying a solid foundation for employment.

## Limitations and future study

The main limitation of this study is that most of the survey participants in the study are engineering students in higher education institutions, who lack an in-depth understanding of the labor market and practical experience at work. Therefore, to increase the statistical power and reliable results, future studies should continue to collect data from this group of participants after they enter the workforce. There is also a need to investigate engineering workers who have already entered the workplace to collect more data and expand the sample size to further validate our findings.

## Data availability statement

The raw data supporting the conclusions of this article will be made available by the authors, without undue reservation.

## Ethics statement

Ethical review and approval was not required for the study on human participants in accordance with the local legislation and institutional requirements. Written informed consent from the patients/participants or patients/participants legal guardian/next of kin was not required to participate in this study in accordance with the national legislation and the institutional requirements.

## Author contributions

JZ contributed to the conception of the study. LX and YD performed the experiment, performed the data analyses, and wrote the manuscript. JZ and YD contributed significantly to analysis and manuscript preparation. GS and WZ helped to perform the analysis with constructive discussions. SP and BG contributed to the revision and various enhancements of the article content. All authors contributed to the article and approved the submitted version.

## Funding

This research is supported by the National Social Science Fund projects of China (no. 20BJY010); National Social Science Fund Post-financing projects of China (no. 19FJYB017); China Sichuan-Tibet Railway Major Fundamental Science Problems Special Fund (no. 71942006); China Qinghai Natural Science Foundation (no. 2020-JY-736); List of Key Science and Technology Projects in China’s Transportation Industry in 2018-International Science and Technology Cooperation Project (nos. 2018-GH-006 and 2019-MS5-100); Emerging Engineering Education Research and Practice Project of Ministry of Education of China (no. E-GKRWJC20202914); Higher Education Teaching Reform Project in Shaanxi Province, China (no. 19BZ016); Humanities and Social Sciences Research Project of the Ministry of Education of China (21XJA752003); Project of the Academy of Social Sciences of Shaanxi Province, China (2022HZ0596); Going Global Partnership: UK-China-ASEAN, Education Partnership Initiative funded by British Council (“Integrated Built Environment Teaching & Learning in the Joint Curriculum Development amid Digital-Driven Industry 4.0 among China, Vietnam, and UK”); International Education Research Program of Chang’an University, China, 2022 (no. 300108221113); and National Natural Science Foundation of China (no. 72074191).

## Conflict of interest

The authors declare that the research was conducted in the absence of any commercial or financial relationships that could be construed as a potential conflict of interest.

## Publisher’s note

All claims expressed in this article are solely those of the authors and do not necessarily represent those of their affiliated organizations, or those of the publisher, the editors and the reviewers. Any product that may be evaluated in this article, or claim that may be made by its manufacturer, is not guaranteed or endorsed by the publisher.
