# Supplementary material for: Assessing the impact of digital education and the role of the big data analytics course to enhance the skills and employability of engineering students
Source: Front Psychol. 2022 Oct 19;13:974574. doi: 10.3389/fpsyg.2022.974574 (PMC9627341; doi:10.3389/fpsyg.2022.974574)
Supplement: Supplementary file 1 [file Data_Sheet_1.docx]

**Appendix**

**Appendix 1：**

Information of big data analytics courses in eight universities

| **University** | **Course code** | **Course name** | **Student Level** |
| --- | --- | --- | --- |
| Columbia University | EECS E6893 | Big Data Analytics | Masters and PhDs |
| Carnegie Mellon University | 67-362/A3 | Big Data Analytics | Undergraduates and Masters |
| Wright State University |  | Big Data Analytics | Undergraduates and Masters |
| Stetson University | CINF 401 | Big Data Mining and Analytics | Undergraduates |
|  | BSAN 398 | Databases and Big Data |  |
| Central South University | 47081202B04 | Data Science and Engineering | Undergraduates and Masters |
|  | 47081202B03 | Data Mining |  |
|  | 47081202D03 | Data Visualization |  |
| University of Chinese Academy of Sciences | 120400MGX005H | Public Big Data Processing and Analysis | Undergraduates and Masters |
| Harbin Institute of Technology | MATH3003 | Big Data Analytics | Undergraduates |
|  | MATH3008 | Data Mining |  |
|  | MATH3007 | Data Visualization |  |
| Xi’an Jiaotong University | 072132 | Big Data Analytics Basics | Maters |
|  | 073010 | Big data analysis and processing methods |  |

**Appendix 2：**

The scale of the impact of big data analytics courses on the six ability dimensions of engineering students

| Variable | Item  labels | Items | Source |
| --- | --- | --- | --- |
| Big data analytics Skills | BDAS1 | I will enforce adequate plans for the introduction and utilization of big data analytics. | (85) |
|  | BDAS2 | I usually perform big data analytics planning processes in systematic and formalized ways. |  |
|  | BDAS3 | I show superior understanding of technological trends. |  |
|  | BDAS4 | I can quickly isolate valuable information from large amounts of data |  |
|  | BDAS5 | I can quickly discover potentially useful knowledge from large amounts of data |  |
| Comunica  -tion Skills | CS1 | I use my feelings to determine how I should communicate. | (118, 119) |
|  | CS2 | Even if I do not receive a clear response from others, I can understand what they intended. |  |
|  | CS3 | When I disagree with someone, I avoid direct conflict. |  |
|  | CS4 | I argue my case to show the merits of my position. |  |
|  | CS5 | I can accept continuous talks |  |
|  | CS6 | I would ask him to explain his meaning in full by asking |  |
| Decision Making  Skill | DMS1 | I feel confident about my ability to makes decisions. | (120) |
|  | DMS2 | I consider how best to carry out a decision. |  |
|  | DMS3 | When making decisions I like to collect a lot of information. |  |
|  | DMS4 | I do not make decisions unless I really have to. |  |
|  | DMS5 | I feel as if I am under tremendous time pressure when making decisions. |  |
| Critical Thinking  Skills | CTS1 | I am able to carefully consider the context of a problem and make a deliberate judgment. | (119) |
|  | CTS2 | I will seek alternative solutions to problems. |  |
|  | CTS3 | I am willing to adopt possible solutions that go beyond existing procedures or regulations. |  |
|  | CTS4 | I can systematically deal with problems and available solutions. |  |
|  | CTS5 | I will seek reasons and evidence to support offered assertions and evaluations. |  |
|  | CTS6 | I can anticipate potential difficulties and suggests possible responses before problems arise. |  |
| Technology  skills | TS1 | I can use big data platforms and tools such as Hadoop, HBase, MongoDB, NoSQL, etc. | (85, 121) |
|  | TS2 | I am very capable in the areas of data and network management and maintenance. |  |
|  | TS3 | I am very capable in terms of programming skills. |  |
| Knowledge  skills | KS1 | I mastered the technical theory of big data analytics, database principles. | (122) |
|  | KS2 | I have improved my learning efficiency and knowledge retention through the big data analytics course. |  |
|  | KS3 | I am able to use big data tools or platforms to search and extract massive amounts of knowledge. |  |

The scale of self‐perceived employability of engineering student

| Knowledge  Capability | KC1 | I will continue to improve my professional knowledge system. | (80) |
| --- | --- | --- | --- |
|  | KC2 | I am able to apply the theoretical knowledge I have learned in practice. |  |
|  | KC3 | I have a wealth of cross-cultural knowledge. |  |
| Lifelong Learning  Capability | LL1 | I expect to go on learning for a long time. | (123) |
|  | LL2 | I like learning new things when I can see how they make sense for me in my life. |  |
|  | LL3 | I like to try out new learning in different ways. |  |
|  | LL4 | Whether in the practicum, classroom or on my own, I am able to follow my own plan of learning. |  |
|  | LL5 | My successes and failures inspire me to continue learning. |  |
| Problem- Solving  Capability | PSC1 | I do not give up solving problems when first attempt fails. | (124) |
|  | PSC2 | I will examine factors contributing to problems. |  |
|  | PSC3 | I will approach problems from many angles. |  |
|  | PSC4 | I will think of as many options as possible. |  |
|  | PSC5 | I believe I can solve a problem if I try hard enough. |  |
| Teamwork  Capability | TC1 | I am actively involved in team activities. | (125) |
|  | TC2 | I can integrate into the team and continuously improve team cohesion. |  |
|  | TC3 | I can find a way to mediate the disagreements and conflicts. |  |
|  | TC4 | When I communicate with others, I listen carefully to each other’s conversations. |  |
|  | TC5 | I can consider other people’s opinions comprehensively in teamwork. |  |
| Innovation  Capability | IC1 | I am good at coming up with innovative ideas. | (126) |
|  | IC2 | I am confident in my creative problem-solving skills. |  |
|  | IC3 | I am good at finding new ways to solve problems. |  |
|  | IC4 | I am able to explore a wide range of knowledge and find my own interests and directions. |  |
|  | IC5 | I constantly question what I have learned and often offer my own unique ideas. |  |
| Leadership  Capability | LC1 | I value completing assignments to the best of my ability over taking advantage of possible loopholes. | (127) |
|  | LC2 | My behavior is pretty consistent from day to day. |  |
|  | LC3 | I usually show a great deal of self-discipline. |  |
|  | LC4 | I build relationships with others at school or work. |  |
|  | LC5 | I develop a positive rapport with my teammates. |  |

**Appendix 3：**

Descriptive statistics and correlation analysis

The descriptive statistics and correlation of studied variables

|  | Mean | SD | 1 | 2 | 3 | 4 | 5 | 6 | 7 | 8 | 9 |
| --- | --- | --- | --- | --- | --- | --- | --- | --- | --- | --- | --- |
| 1. Knowledge skill | 3.314 | 0.895 | 0.895 |  |  |  |  |  |  |  |  |
| 2. Decision-making skill | 3.695 | 0.479 | 0.284^***^ | 0.710 |  |  |  |  |  |  |  |
| 3. Critical thinking skill | 3.830 | 0.558 | 0.083 | 0.623^***^ | 0.806 |  |  |  |  |  |  |
| 4. Technology skill | 2.346 | 0.990 | 0.585^***^ | 0.200^*^ | 0.140 | 0.925 |  |  |  |  |  |
| 5. Big data analytics skill | 2.800 | 0.867 | 0.573^***^ | 0.163^*^ | 0.096 | 0.738^***^ | 0.890 |  |  |  |  |
| 6. Communication skill | 3.675 | 0.574 | 0.237^**^ | 0.233^**^ | 0.428^***^ | 0.213^**^ | 0.302^***^ | 0.753 |  |  |  |
| 7. Human capital | 3.727 | 0.555 | 0.314^**^ | 0.527^**^ | 0.676^**^ | 0.330^**^ | 0.368^**^ | 0.576^**^ | 0.806 |  |  |
| 8. Individual attributes | 3.809 | 0.508 | 0.312^***^ | 0.610^***^ | 0.682^***^ | 0.261^**^ | 0.282^***^ | 0.447^***^ | 0.806^***^ | 0.773 |  |
| 9. Career development | 3.769 | 0.540 | 0.339^***^ | 0.591^***^ | 0.613^***^ | 0.299^***^ | 0.288^***^ | 0.397^***^ | 0.707^***^ | 0.790^***^ | 0.796 |

Note: The coefficients represent in the diagonal are the square root value of AVE. SD=standard deviation. * *p* <0.05, ** *p* < 0.01, *** *p* < 0.001.

**Appendix 4:**

Results of CITC and alpha-coefficient analysis of study variables

| Variable | Dimension | Corrected Item and Total Correlation | Alpha |
| --- | --- | --- | --- |
| KS1 | KS | .491 | 0.875 |
| KS2 |  | .490 |  |
| KS3 |  | .387 |  |
| DMS1 | DMS | .569 | 0.740 |
| DMS2 |  | .511 |  |
| DMS3 |  | .565 |  |
| DMS4 |  | .252 |  |
| DMS5 |  | .209 |  |
| CTS1 | CTS | .570 | 0.889 |
| CTS2 |  | .529 |  |
| CTS3 |  | .538 |  |
| CTS4 |  | .583 |  |
| CTS5 |  | .551 |  |
| CTS6 |  | .525 |  |
| TS1 | TS | .473 | 0.915 |
| TS2 |  | .485 |  |
| TS3 |  | .491 |  |
| BDAS1 | BDAS | .462 | 0.934 |
| BDAS2 |  | .455 |  |
| BDAS3 |  | .530 |  |
| BDAS4 |  | .513 |  |
| BDAS5 |  | .505 |  |
| CS1 | CS | .389 | 0.846 |
| CS2 |  | .458 |  |
| CS3 |  | .337 |  |
| CS4 |  | .475 |  |
| CS5 |  | .516 |  |
| CS6 |  | .424 |  |
| KC1 | KC | .609 | 0.817 |
| KC2 |  | .620 |  |
| KC3 |  | .566 |  |
| LL1 | LL | .612 | 0.898 |
| LL2 |  | .630 |  |
| LL3 |  | .605 |  |
| LL4 |  | .648 |  |
| LL5 |  | .622 |  |
| TC1 | TC | .509 | 0.866 |
| TC2 |  | .598 |  |
| TC3 |  | .614 |  |
| TC4 |  | .549 |  |
| TC5 |  | .550 |  |
| IC1 | IC | .581 | 0.917 |
| IC2 |  | .653 |  |
| IC3 |  | .631 |  |
| IC4 |  | .622 |  |
| IC5 |  | .676 |  |
| PSC1 | PSC | .692 | 0.891 |
| PSC2 |  | .697 |  |
| PSC3 |  | .764 |  |
| PSC4 |  | .639 |  |
| PSC5 |  | .607 |  |
| LC1 | LC | .653 | 0.847 |
| LC2 |  | .601 |  |
| LC3 |  | .635 |  |
| LC4 |  | .632 |  |
| LC5 |  | .606 |  |

Note: CITC=Corrected Item-Total Correlation

**Appendix 5:**

| Dimension | KMO | P |
| --- | --- | --- |
| KS | 0.671 | < 0.001 |
| DMS | 0.569 | < 0.001 |
| CTS | 0.880 | < 0.001 |
| TS | 0.713 | < 0.001 |
| BDAS | 0.813 | < 0.001 |
| CS | 0.837 | < 0.001 |
| KC | 0.672 | < 0.001 |
| LL | 0.842 | < 0.001 |
| TC | 0.819 | < 0.001 |
| IC | 0.869 | < 0.001 |
| PSC | 0.862 | < 0.001 |
| LC | 0.799 | < 0.001 |

Note: KMO= Kaiser-Meyer-Olkin
